# Antibiotics therapy combined with probiotics administered intravaginally for the treatment of bacterial vaginosis: A systematic review and meta-analysis

**DOI:** 10.1515/med-2023-0644

**Published:** 2023-09-13

**Authors:** Siyu Ma, Wei Wang, Yanli Su, Wei Sun, Liyan Ma

**Affiliations:** Department of Clinical Laboratory, Beijing Friendship Hospital, Capital Medical University, Beijing, 100050 China; Department of Infection and Critical Care Medicine, Beijing Friendship Hospital, Capital Medical University, Beijing, China

**Keywords:** vaginitis, metronidazole, clindamycin, lactobacilli, recurrence rate, cure rate

## Abstract

The objective was to examine the pooled effects of antibiotic–probiotic combinations by examining the cure rate and recurrence rate for bacterial vaginosis (BV). A systematic literature search was conducted from electronic databases. All parallel randomized controlled trials (RCTs) that focused on the effects of antibiotics combined with intravaginal probiotics were included. Cure rate and recurrence rate were the primary and secondary outcomes to be analyzed. Meta-analysis was conducted following the Cochrane handbook for Systematic Reviews of Interventions. As a result, of 923 studies identified, 11 articles involving 1,493 BV patients met the inclusion criteria and nine were available for meta-analysis. A meta-analysis of two studies evaluated the recurrence rate 12–16 weeks after treatment. Results showed a statistically significant difference favoring the antibiotics plus probiotics group vs the antibiotics plus placebo group (relative risk 0.62, 95% confidence interval [CI]: 0.45–0.85). The narrative review in one study indicated that the cure rate was higher in the antibiotics plus probiotics group, giving a significant HR ratio of 0.73 (95% CI 0.54–0.98) (*p* = 0.042). In conclusion, vaginal application of *Lactobacillus* in combination with antibiotics for the treatment of BV could be a promising method for both reducing the recurrence rate and relieving symptoms of BV.

## 1 Introduction

Bacterial vaginosis (BV) is a common vaginal infectious disease caused by the decrease or disappearance of lactobacilli and the increase of facultative anaerobes and anaerobes. Under antibiotic treatment, such as nitroimidazoles (metronidazole and tinidazole) and clindamycin, the BV recurrence rate remains high at up to 80% [1]. BV is associated with an increased risk of pelvic inflammatory disease, post-surgical infection, adverse pregnancy outcomes, and sexually transmitted diseases. The incidence of BV varies in different countries and regions owing to different populations, races, and diagnostic methods, ranging from 7.1 to 29.2% in North America, 7.0 to 23.2% in Western Europe, 16.2 to 50.0% in the Middle East, and 10.3 to 32.5% in South and Southeast Asia [2]. In Africa, this rate is 29.9–52.4% [3]. Survey data in China show that BV is present in around 11.0% of women undergoing physical examination [4] and 36.0–60.0% of patients with vaginal inflammation in gynecological clinics [5,6,7]. Currently, BV diagnosis is mostly based on Amsel clinical diagnostic criteria and Gram-staining Nugent score diagnostic criteria [8,9,10]. Antibiotics alone are not satisfactory in treating BV, and for recurrent BV, there is no accepted optimal management plan. *Lactobacillus* preparations provide a new option for the treatment of BV [11]. A review published in 2020 discovered that *Lactobacillus* had a positive influence on immunomodulation and restoration of healthy microflora in the gut and vagina. It also indicated that *Lactobacillus* had beneficial effects in reducing the recurrence rate of vaginal infection and preventing vaginally-acquired infections [12].

At present, the administration of probiotics is mainly by mouth [13]. Theoretically, vaginal administration of probiotics could allow a more direct, quicker, and targeted colonizing action to restore the altered vaginal microbiota. A systematic review published in 2021 indicated that vaginal probiotics moderately modulated the relative abundance of abnormal microbiota, coinciding with an increase in *Lactobacillus* species [14].

In recent years, studies have shown that antibiotics and vaginal probiotics are effective in improving the cure rate and reducing the recurrence rate of BV [15,16]. However, a systematic review of research in this field is still lacking. In particular, for recurrent BV, it is not known whether antibiotics combined with vaginal probiotics provide a more effective treatment. The purpose of this systematic review was to make a systematic evaluation and meta-analysis of the current studies on antibiotics plus vaginal use of probiotics for BV, so as to clarify the value of this combination of medications and provide a basis for clinicians’ decision-making and further research.

## 2 Methods

### 2.1 Data sources and searching strategies

This systematic review has been registered in the International Prospective Register of Systematic Reviews (PROSPERO), the registration number was CRD42014015079. Preferred Reporting Items for Systematic Reviews and Meta-Analyses (PRISMA) statement guidelines were followed in the construction of this systematic review [17]. A comprehensive search was conducted in the following electronic databases from their inception to August 2021: The Cochrane Central Register of Controlled Trials in the Cochrane Library, the Cochrane Library of Systematic Reviews, MEDLINE/PubMed, and EMBASE. Reference lists of retrieved articles were also screened for eligible literature. Searches were limited to articles published in English and conducted on humans. Table 1 presents the search strategy for MEDLINE.

**Table 1:** MEDLINE search strategy

	Search items
1	RCT
2	Controlled clinical trial
3	Randomized
4	Trial
5	or/1–4
6	Bacterial vaginosis or BV/
7	Bacterial vaginitis or BV/
8	or/6–7
9	Drug therapy/
10	Treatment/
11	Antibiotics/
12	or/9–11
13	Probiotics/
14	*Lactobacillus*/
15	or/13–14
16	5 and 8 and 12 and 15

### 2.2 Inclusion and exclusion criteria

We included only parallel RCTs. Studies that did not provide sufficient data for extraction or calculations were excluded.

A participants, interventions, comparators, outcome measures framework was used to determine the eligibility for study inclusion.

✓ Participants: Patients diagnosed with BV, with or without symptoms, based on Amsel’s criteria or the Nugent score.
✓ Interventions: Probiotics administered intravaginally in conjunction with antibiotic therapy, oral or intravaginal.
✓ Comparators: No treatment, placebo, or a different probiotic/antibiotic type or probiotic/antibiotic dose.
✓ Outcome measures: The primary outcome was the BV cure rate. The secondary outcome was the recurrence rate of BV, defined as the presence of ≥3 for Amsel’s criteria or a Nugent score ≥7.

### 2.3 Selection of studies

**Figure 1:**
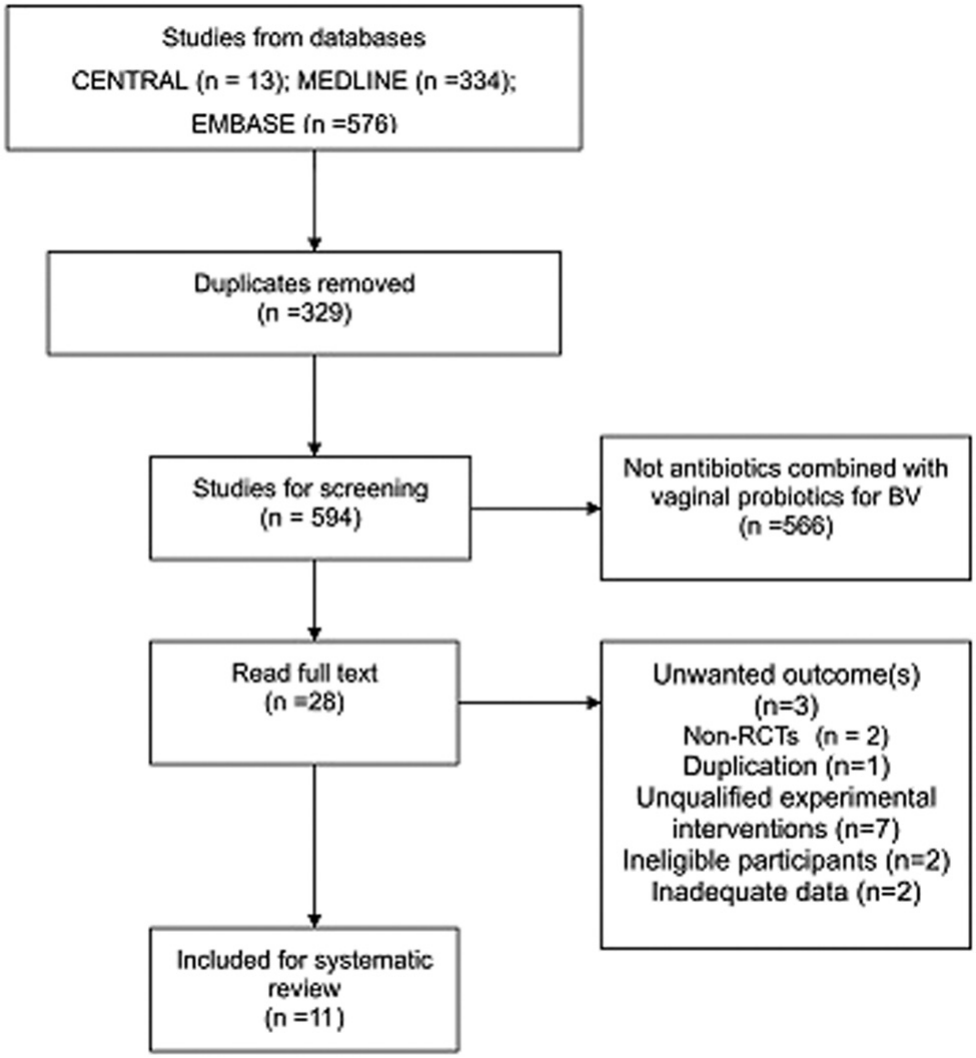
PRISMA flow diagram.

S.M. and W.W. independently screened the search results by reading through titles and abstracts. After removing duplicates and ineligible articles, the reviewers read the full texts to determine whether they were able to be included. Studies were excluded if participants used antibiotics or probiotics solely or were co-infected with other sexually transmitted infections. Discrepancies were resolved by a third reviewer, L.M. Study selection is summarized in a PRISMA flow diagram (Figure 1).

### 2.4 Risk of bias assessment

W.S. and L.M., two independent reviewers, used the Cochrane Risk of Bias tool to assess the risk of bias. The sources of bias included: selection bias (random sequence generation and allocation concealment); performance bias (blinding of participants and personnel); detection bias (blinding of outcome assessment); attrition bias (incomplete outcome data); and reporting bias (selective outcome reporting). The risk of bias was rated using predetermined criteria as follows: low, high, or unclear. As a result, one of the included studies indicated a low risk of bias, three of the studies were considered high risk of bias, and the other seven were unclear (some concerns) of risk of bias.

### 2.5 Data extraction and management

W.W. and S.M., two review authors, independently assessed and extracted the study data using a data extraction form that covered basic details, participant details, diagnostic procedures (Amsel’s criteria or Nugent score), intervention details (genus of the probiotics and dose and duration of the probiotics and antibiotics), and outcome measures (cure rates of BV, recurrences rates of BV, vaginal lactobacilli colonization, restoration of a normal vaginal microbiota, and occurrence of adverse effects). Extracted data were checked by W.S. Disagreements were resolved through discussion. In case further information or clarification was needed, the corresponding author of the original article was contacted through email.

### 2.6 Data synthesis

We used the RevMan Analyses statistical package in Review Manager (version 5.3) (Copenhagen: The Nordic Cochrane Centre, The Cochrane Collaboration, 2014). For dichotomous outcomes, we derived the relative risk (RR) and 95% confidence intervals (CI) for each study. Where there is heterogeneity (*I* ^2^ > 75%), a random-effect model was used.

We also provided a narrative review for studies that cannot be meta-analyzed.

### 2.7 Confidence in cumulative evidence

In order to describe the strength of evidence bodies acquired from meta-analysis, we used the Grading of Recommendation Assessment, Development and Evaluation (GRADE) system to assess bias risk, consistency, directness, precision, and publication bias [18]. The quality of the evidence bodies was identified as high (the true effect lies close to that of the estimate of the effect), moderate (the true effect is likely to be close to the estimate of the effect, but there is a possibility that it is substantially different), low (the true effect may be substantially different from the estimate of the effect), or very low (the true effect is likely to be substantially different from the estimate of effect).

## 3 Results

### 3.1 Identification of studies

A total of 923 studies were obtained by searching established databases. After removing 329 duplicates, we read and screened 594 titles and abstracts. In the next step, we excluded 566 articles based on the title and abstract, because their research content did not conform to this review. The full texts of 28 studies were downloaded and screened based on the predefined inclusion criteria. Altogether 17 studies were excluded for the following reasons: three studies did not report data on the outcome(s) of interest [15,16,19], two studies were shown to be non-RCTs [20,21], one study was considered to be a duplication of a former publication [22], seven studies introduced unqualified experimental interventions such as oral probiotics [23–27] and complementary medicine [28], two studies focused on ineligible participants (vaginal infections instead of BV) [29,30], and two studies did not provide adequate data and did not reply to our inquiry emails [31,32]. Finally, 11 studies were included [33–43]. We also screened the reference lists of included studies, which located 38 trials. No additional studies were included. The reasons for exclusion covered duplication, disqualification of study type, participants, and interventions. The literature screening process of databases is shown in Figure 1.

### 3.2 Description of included studies

A total of 1,493 BV patients were involved in this systematic review. The publication year of the included studies ranged from 2005 to 2020. The sample size of the included studies ranged from 30 in South Africa [36] to 450 in Australia [33]. Two studies were three-armed [33,37]; others were two-armed [34–36,38–43].

Five studies compared antibiotics plus probiotics administered sequentially vs antibiotics plus placebo administered sequentially [33–35,40,41]. Four studies compared antibiotics plus probiotics administered sequentially vs antibiotics only [36–39]. In the control group, participants did not have any treatment after completion of antibiotics courses. The above research data were combined for meta-analysis.

The three-armed study also compared oral metronidazole plus probiotics administered sequentially vs oral metronidazole plus vaginal clindamycin administered sequentially [37]. One study compared antibiotics plus probiotics administered simultaneously vs antibiotics plus placebo administered simultaneously [42]. One study compared antibiotics plus continuous probiotics (once daily) administered sequentially vs antibiotics plus interrupted use of probiotics (twice a week) administered sequentially [43]. A narrative review was done for these studies.

Antibiotics used in the experimental group included metronidazole [33,34,36,38,41,42], clindamycin [35,39,40,43], and a combination of three oral antibiotics: cefixime, doxycycline, and metronidazole [37]. Of the included 11 studies, two used *Lactobacillus crispatus* for the treatment or prevention of BV and nine used non-*L. crispatus* strains. The characteristics of the included studies are shown in Table A1.

### 3.3 Risk of bias evaluation results

**Figure 2:**
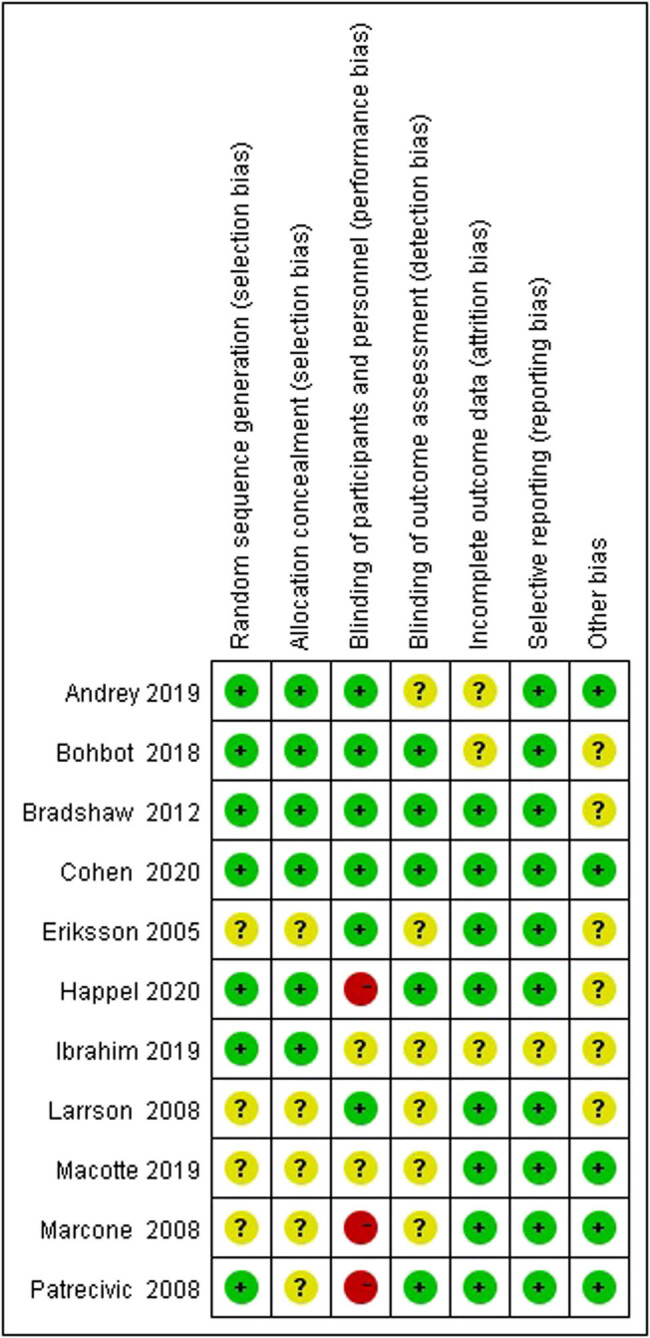
The risk of bias summary of included studies.

Figure 2 shows the risk of bias. For studies that used a non-placebo control, it was not possible to blind patients; therefore, these studies were considered to have a high risk of performance bias [36,38,39]. In addition, the most common factors leading to study quality degradation were selection bias, which meant that some included studies did not describe any form of allocation concealment [35,37–40], and detection bias, which meant that some studies did not clarify whether the outcome assessors were blinded [35,37,38,40,42,43].

### 3.4 Meta-analysis

#### 3.4.1 Antibiotics + probiotics (sequentially) vs antibiotics + placebo (sequentially)

##### 3.4.1.1 Short-term cure rate (4–8 weeks)

**Figure 3:**
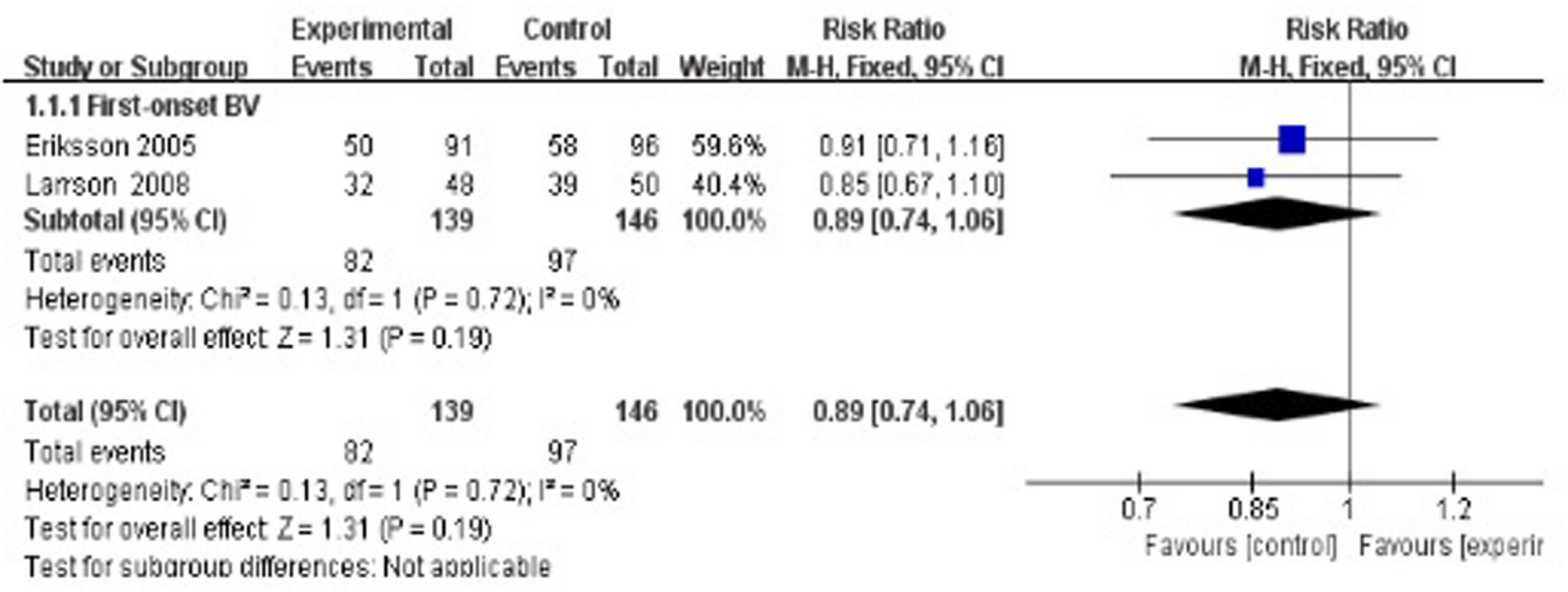
Short-term cure rate of antibiotics + probiotics (sequentially) vs antibiotics + placebo (sequentially).

Two studies evaluated the cure rate at 4–8 weeks after treatment [35,40], suggesting no statistically significant difference between the treatment group and the control group (RR 0.89, 95% CI 0.74–1.06; Figure 3).

##### 3.4.1.2 Middle-term recurrence rate (12–16 weeks)

**Figure 4:**
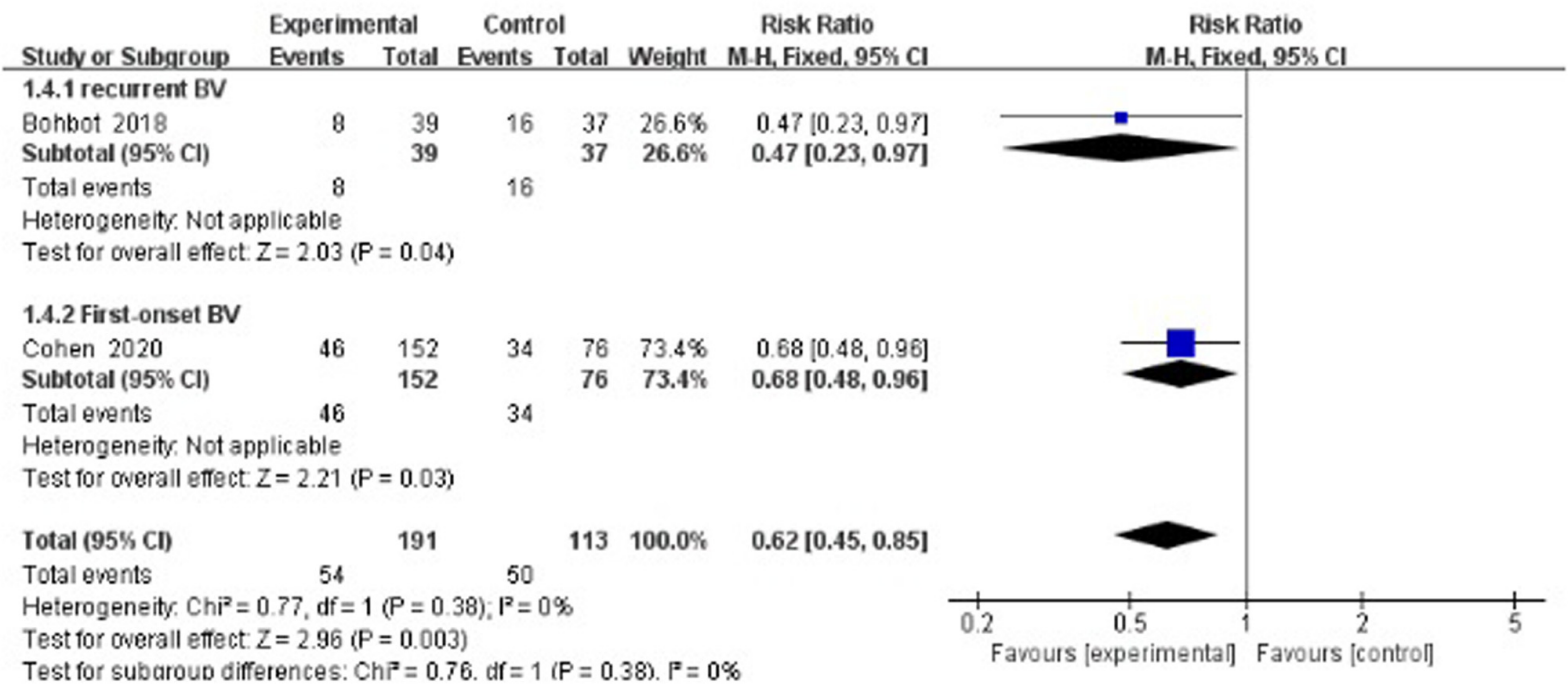
Middle-term recurrence rate of antibiotics + probiotics (sequentially) vs antibiotics + placebo (sequentially).

Two studies evaluated the recurrence rate at 12–16 weeks after treatment [34,41]. The results showed a statistically significant difference between the treatment group and the control group (RR 0.62, 95% CI 0.45–0.85). Here, this meta-analysis contained one study that focused on first-onset BV^36^ and one study on recurrent BV [41]. As a single study, the Bohbot study did not show positive results (RR 0.47, 95% CI 0.23–0.97), whereas the Cohen study had a result favoring the experimental group (RR 0.68, 95% CI 0.48–0.96; Figure 4).

##### 3.4.1.3 Long-term recurrence rate (24 weeks)

**Figure 5:**
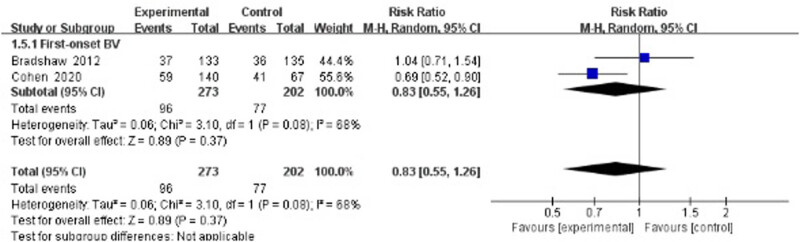
Long-term recurrence rate of antibiotics + probiotics (sequentially) vs antibiotics + placebo (sequentially).

Two studies evaluated the recurrence rate 24 weeks after treatment [33,34] and suggested no statistically significant difference between the treatment group and the control group (RR 0.83, 95% CI 0.55–1.26; Figure 5).

#### 3.4.2 Antibiotics + probiotics (sequentially) vs antibiotic-only short-term cure rate (4 weeks)

**Figure 6:**
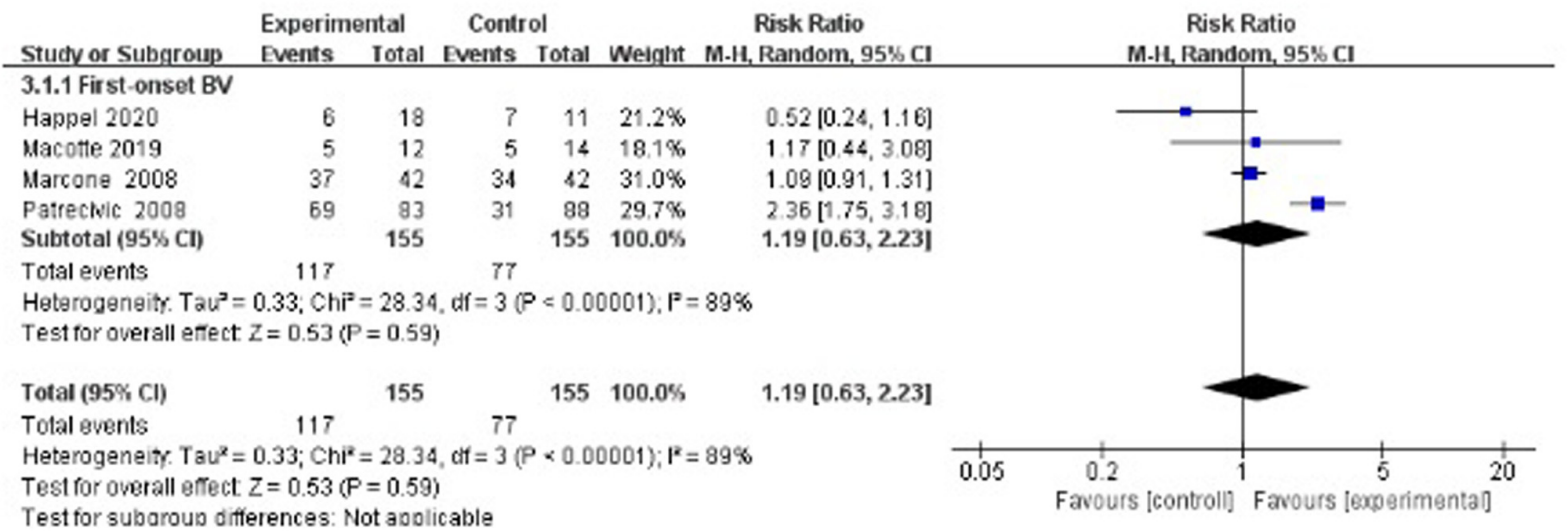
Short-term cure rate of antibiotics + probiotics (sequentially) vs antibiotics only.

Four studies evaluated the cure rate 4 weeks after treatment [36–39], suggesting no statistically significant difference between the treatment group and the control group (RR 1.19, 95% CI 0.63–2.23; Figure 6).

##### 3.4.2.1 Long-term cure rate (24 weeks)

**Figure 7:**
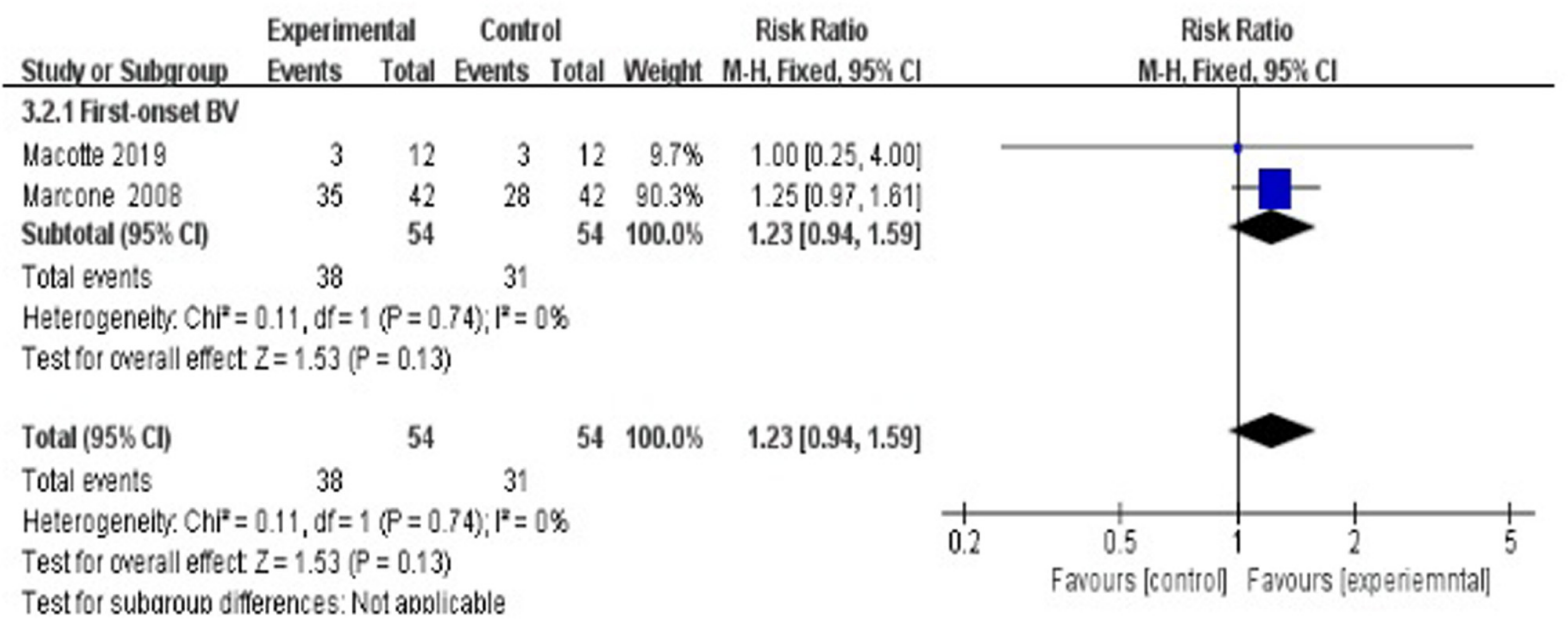
Long-term cure rate of antibiotics + probiotics (sequentially) vs antibiotics only.

Three studies evaluated the cure rate at 24 weeks after treatment [35,37,38], suggesting a statistically significant difference between the treatment group and the control group (RR 1.23, 95% CI 0.94–1.59; Figure 7).

### 3.5 Narrative analysis

Larsson’s study comparing antibiotics plus probiotics sequentially vs antibiotics plus placebo sequentially had a follow-up duration of six menstrual cycles [35]. At the end of the study, 64.9% (24/37) of first-onset BV patients in the experimental group were pronounced cured compared to 46.2% (18/39) of the placebo group, giving a significant HR ratio of 0.73 (95% CI: 0.54–0.98; *p* = 0.042).

Bradshaw’s study compared oral metronidazole plus probiotics sequentially vs oral metronidazole plus vaginal clindamycin cream sequentially [33]. Short-term (4 weeks) and long-term (24 weeks) recurrence rates for first-onset BV patients were 9/133 vs 5/140 and 37/133 vs 42/140, respectively. Both recurrence rates were negative in inter-group comparisons at 23% (95% CIs 19–27) and 54% (95% CIs 49–59), respectively (*p* = 0.87).

Sgibnev’s study compared antibiotics plus probiotics administered simultaneously vs antibiotics plus placebo administered simultaneously for BV patients with *Trichomonas vaginalis* (TV) [42]. Observation in this study was for a short term until 15 days after treatment. Results indicated that the combined use of probiotics and metronidazole reduced BV symptoms more effectively in comparison with the placebo at time points of the first stage (8 days) and second stage (15 days).

Elsharkawy’s study compared antibiotics plus continuous vaginal probiotics (once daily) sequentially vs antibiotics plus interrupted vaginal probiotics (twice a week) sequentially [43]. At the initial visit, 4 weeks after treatment, there was no significant difference in cure rate between the continuous probiotics group and interrupted probiotics group (87.4 vs 82.5%; *p* = 0.81). Furthermore, there was no significant difference between the two groups in the recurrence rate at 1-, 3-, 6-, and 9-month follow-up visits (*p* = 0.16, *p* = 0.42, *p* = 0.59, *p* = 0.66).

Happel’s study evaluated the recurrence rate between antibiotics plus probiotics sequentially vs antibiotics only at 24 weeks after treatment [36], suggesting no statistically significant difference between the treatment group and the control group (RR 0.78, 95% CI 0.19–3.21).

### 3.6 GRADE evaluation results for evidence bodies from meta-analysis

**Figure 8:**
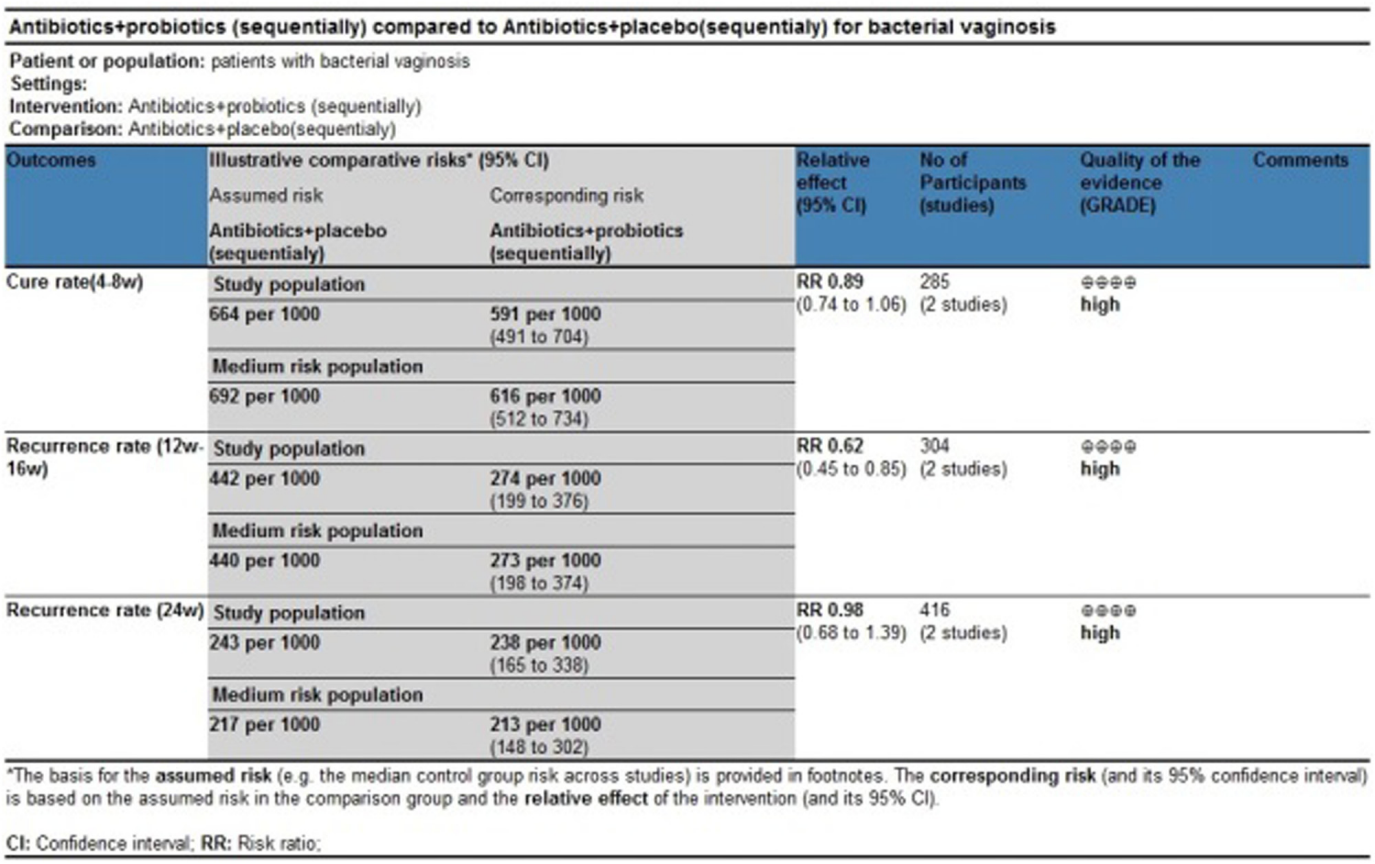
GRADE evaluation of evidence body on antibiotics + probiotics (sequentially) vs antibiotics + placebo (sequentially).

**Figure 9:**
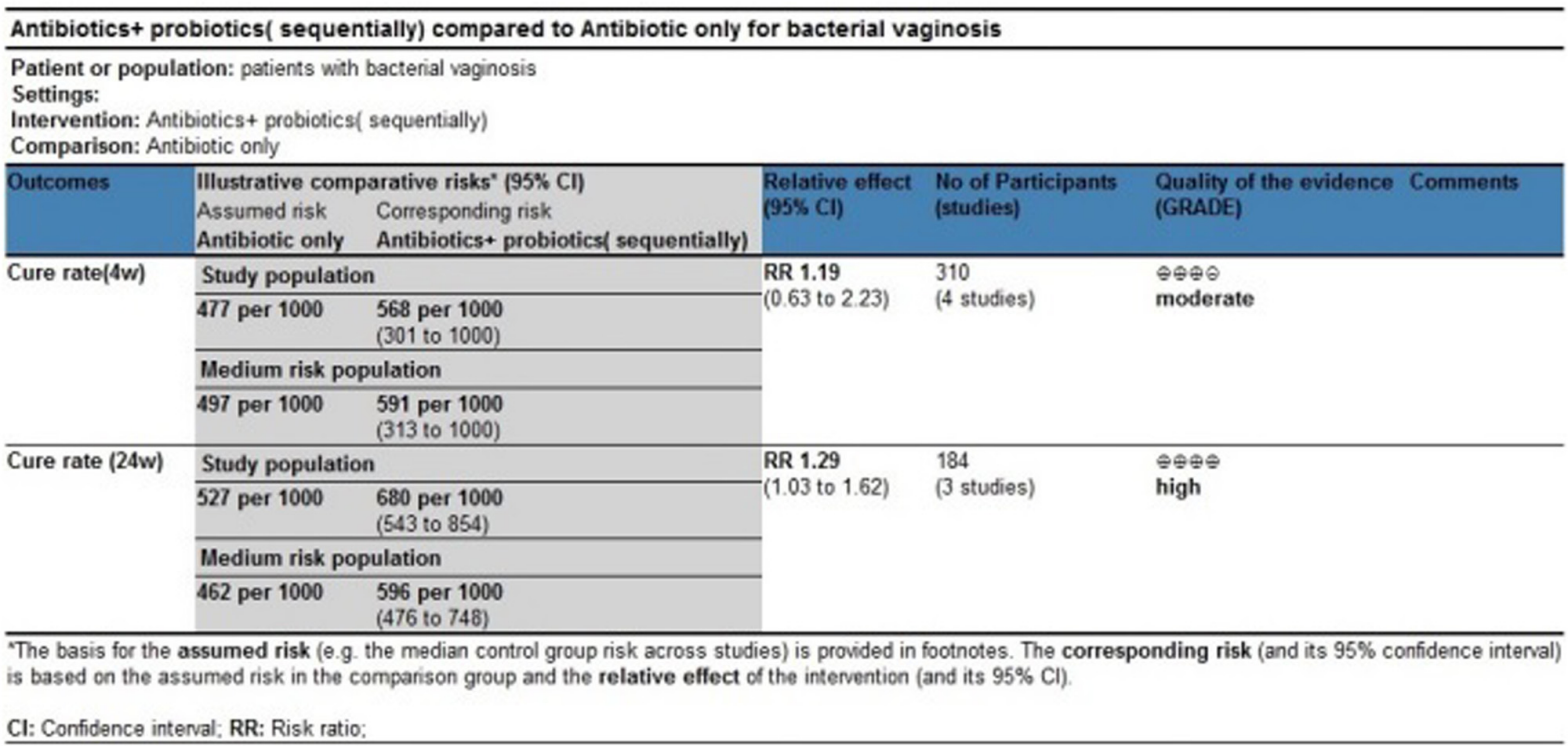
GRADE evaluation of evidence body on antibiotics + probiotics (sequentially) vs antibiotics only.

Most of the evidence bodies generated after the combination of studies were of high quality according to GRADE evaluation, while a few of them were of medium quality. No evidence bodies of low or very low quality were produced. The main results of the GRADE evaluation are shown in Figures 8 and 9.

## 4 Discussion

Although the use of antibiotics in combination with probiotics for BV has entered active clinical research, its true clinical value, specifically for antibiotics in combination with probiotics used vaginally, has not been fully evaluated to date. Two systematic reviews published in the past 2 years involved antibiotics in combination with probiotics to a limited extent [44,45], but neither had any restriction placed on probiotic administration. In addition, one study took BV and vulvovaginal candidiasis as a combined group of participants [44]. The oral route has been used and studied more often for treating BV despite a need for further transference to the colonization site. However, survival through the low pH of the upper gastrointestinal region may block the efficient transfer of oral probiotics. On the contrary, probiotics administered vaginally may control the recolonization of *Lactobacillus* without any transfer needs or survival concerns in treating vulvovaginal infections [46]. Oral and vaginal administrations, regardless of the continuous discussion, should be considered as a clinical heterogeneity in meta-analysis. To the best of our knowledge, the present review is the first to take this into account.

Probiotics have been verified as a safe and highly effective adjunctive therapy for the treatment of antibiotic-associated diarrhea and recurrent urinary tract infections [47,48]. In terms of the vagina, lactobacilli are commonly used as probiotics for their ability to inhibit the growth of pathogens and the production of lactic acid and H_2_O_2_. Two trials (Bohbot and Cohen) in our meta-analysis report very promising results [34,41]. Middle-term recurrence rate (12–16 weeks) was significantly reduced after metronidazole combined with *L. crispatus* intravaginal administration compared with metronidazole plus placebo. As Cohen et al. presented, patients receiving vaginal *L. crispatus* CTV-05 for 10 weeks had a lower BV recurrence rate (30%) at 12 weeks following the initial treatment of metronidazole [34]. In another study (Bohbot et al.), *L. crispatus* was given for 4 weeks immediately after oral metronidazole, resulting in a significantly lower BV recurrence rate at 16 weeks compared with the placebo-control group (20.5% vs 43.2%) [41]. However, as shown in Figure 5, the 24 week follow-up did not reveal any difference in recurrence rate. Though Cohen et al. reported a protective effect of the intervention group at 24 weeks, Bradshaw’s study showed that there was no benefit from combining oral metronidazole with 12 days of vaginal *Lactobacillus acidophilus* [33,34]. This lack of combined significance may be explained by the difference in courses and strains of *Lactobacillus* administration, the heterogeneity of behavioral characteristics, and the compliance of participants over the long-term (24 weeks) follow-up period.

Duration of follow-up seemed to be a factor associated with the cure rate. No matter whether antibiotic plus placebo or antibiotic only was used as the control group, short-term (4 weeks) cure rates of antibiotics plus probiotics were overall negative in comparison, as shown in Figures 3 and 6. In these six studies, after antibiotic treatment finished, the experimental group received vaginal capsules or tampons containing mainly *Lactobacillus* from 10 days (or 8–10 times) to 6 months. With the exception of Petricevic’s study, the remaining studies showed no improvement after antibiotic plus probiotic treatment. A limitation was that these studies assessed the short-term (4 weeks) cure rate immediately after probiotic use. Fortunately, during the long-term follow-up (24 weeks) of Larsson’s study, the cure rate of the experimental group was significantly improved compared with the placebo group (64.9% vs 46.2%, *p* = 0.042) [35]. Therefore, we speculated that a sufficient time for the vaginal colonization of extraneous lactobacilli to reconstruct the normal vaginal flora is essential for the assessment of the therapeutic effect.

Figure 7 shows that the long-term (24 weeks) cure rate was not significantly higher in the experimental group (antibiotics combined with probiotics) than in the control group (antibiotic only). In Marcone’s study, although the difference was not statistically significant (*p* = 0.07), the results still indicated that vaginal colonization by *Lactobacillus rhamnosus* took better control of BV [38]. In another study from Macotte et al., after a single oral dose of metronidazole, the 6-month BV cure rates were similar between the antibiotic plus probiotic group (3/12, 25%) and antibiotic-only group (3/12, 25%) [37]. There may be two reasons for this negative result: (i) It is possible that the sample size of these included studies was ultimately not large enough to detect statistically significant differences between the treatment groups; (ii) Macotte et al.’s study used metronidazole in a single oral dose, which possibly led to a lower cure rate than the generally recommended 7-day course of metronidazole. Compared with the subgroup of antibiotics plus probiotics (sequentially) vs antibiotic-only, a placebo-controlled study from Larsson et al. showed a significantly high long-term cure rate in the experimental group. Participants were not given any trade names or information to enable them to link a product with its appearance or duration of therapy, which would bring a subject-expectancy effect to the participants in the antibiotics plus placebo group.

In the three-armed study, Bradshaw et al. compared the recurrence rate of oral metronidazole plus probiotics sequentially vs oral metronidazole plus vaginal clindamycin cream sequentially at 4 weeks and 24 weeks. Results showed that the use of metronidazole combined with lactobacilli or clindamycin had a similar recurrence rate. The advantage is that the administration of lactobacilli could reduce the use of antibiotics. Furthermore, a combination of two different categories of antibacterial is not included in the current treatment guidelines suggested by the Centers for Disease Control.

There seems to be a consensus that antibiotics and probiotics should be used sequentially instead of simultaneously. In contrast to metronidazole, clindamycin is a broad-spectrum antibiotic that inhibits the growth of normal flora and might increase the vaginal reservoir of macrolide-resistant bacteria. However, the administration frequency of probiotics was to be unified. Elsharkawy’s study indicated continuous probiotics (once daily) and interrupted vaginal probiotics (twice a week) had a similar short-term cure rate and recurrence rate up to 9 months [43]. This may provide an economic choice for undeveloped areas.

Cohen et al. reported that recurrence of BV occurred in 30% of participants, and *L. crispatus* CTV-05 was detected in 79% of participants in the probiotic group at 12 weeks [34]. This indicated that the higher the vaginal colonization with *L. crispatus*, the better the prevention effect for recurrence. Therefore, vaginal probiotics may be the dawn of future research that will lead to more efficient ways of exogenous lactobacilli colonization in the vagina as vaginal microbiome transplants (VMTs). In five case series presented by Lev-Sagie et al., VMT was associated with full long-term remission until the end of follow-up at 5–21 months after VMT, defined as a marked improvement of symptoms according to Amsel criteria; the appearance of microscopic vaginal fluid and restore of a *Lactobacillus*-dominated vaginal microbiome [49].

There were two limitations in our research. One, no studies conducted in Asia or South America were included. Two, no subgroup/sensitivity or publication bias analysis was carried out because of the low number of included studies.

## 5 Conclusions

In conclusion, the vaginal application of lactobacilli after administration of antibiotics for the treatment of BV could be a promising method both for reducing the risk of recurrence of BV and for reducing symptoms. Therefore, lactobacilli may be helpful in improving the reproductive health of women. Further well-designed and larger trials are needed to determine factors including probiotic strain selection and dose/frequency of administration.

## Appendix

**Table A1:** Characteristics of 11 included studies in the systematic review of antibiotics therapy combined with probiotics administered intravaginally for the treatment of BV

No.	Author (year of publication)	Participants	Sample size			Interventions and durations			Outcome measures and follow-up periods (weeks after therapy)
			E	C1	C2	E	C1	C2	
1	Bradshaw (2012) [33]	First-onset BV	150	150	150	Oral metronidazole 400 mg twice daily for 7 days plus (sequentially) probiotics contained at least 107 colony-forming units (CFU) of live *L. acidophilus* KS400, 0.03 mg estriol, and excipients	Oral metronidazole 400 mg twice daily for 7 days plus (sequentially) placebo (a single vaginal pessary) for 12 nights	Oral metronidazole 400 mg twice daily for 7 days plus (sequentially) vaginal 2% clindamycin cream in a plain white tube for 7 nights	Recurrence rate (4 weeks)
									Recurrence rate (24 weeks)
2	Cohen (2020) [34]	First-onset BV	152	76		Vaginal 0.75% metronidazole gel for 5 days plus (sequentially) four consecutive daily doses of Lactin-V during week 1, followed by twice weekly doses for 10 weeks	Vaginal 0.75% metronidazole gel for 5 days plus (sequentially) four consecutive daily doses of placebo during week 1, followed by twice weekly doses for 10 weeks		Recurrence rate (12–16 weeks)
3	Larsson (2008) [35]	First-onset BV	50	50		2% Vaginal clindamycin cream for 7 days plus (sequentially) vaginal gelatin capsules containing 108–109 freeze-dried lactobacilli for 10 days or until menstruation commenced. The treatment with vaginal lactobacilli capsules was repeated for three cycles	2% Vaginal clindamycin cream for 7 days plus (sequentially) placebo capsules of identical appearance for 10 days or until menstruation commenced. The treatment with placebo was repeated for three cycles		Cure rate (4–8 weeks)
									Cure rate (24 weeks)
4	Happel (2020) [36]	First-onset BV	18	12		0.75% Metronidazole gel, 5 g vaginally, once a day for 5 days plus 10 days of oral capsules together with twice daily vaginal spray, which contained lyophilized *L. acidophilus*, *L. rhamnosus* GG, *Bacillus bifidum*, and *Bacillus longum* at ≥2 × 10^9^ CFU	0.75% Metronidazole gel, 5 g vaginally, once a day for 5 days only		Cure rate(4 weeks)
									Recurrence rate (24 weeks)
5	Marcotte (2019) [37]	First-onset BV (healthy for C2)	12	14	13	Cefixime (400 mg stat), doxycycline (100 mg twice daily for 7 days) and metronidazole (2 g stat) plus (sequentially) self-administered probiotic capsules containing probiotic strains *L. gasseri* DSM 14869 and *L. rhamnosus* DSM 14870 at 1 × 108 CFU of each strain/capsule vaginally once daily for 30 days thereafter once a week until Day 190	Cefixime (400 mg stat), doxycycline (100 mg twice daily for 7 days) and metronidazole (2 g stat) only	Self-administered probiotic capsules containing probiotic strains *Lactobacillus gasseri* DSM 14869 and *L. rhamnosus* DSM 14870 at 1 × 108 CFU of each strain/capsule vaginally once daily for 30 days thereafter once a week until Day 190	Cure rate (4 weeks)
									Cure rate (24 weeks)
									Recurrence rate (24 weeks)
6	Marcone (2008) [38]	First-onset BV	42	42		Oral metronidazole 500 mg twice a day for 7 days plus (sequentially) vaginal application (one tablet containing 40 mg, i.e., >40,000 CFU) of freeze-dried *L. rhamnosus* once a week at bedtime for 2 months	Oral metronidazole 500 mg twice a day for 7 days only		Cure rate (4 weeks)
									Cure rate (24 weeks)
7	Petricevic (2008) [39]	First-onset BV	95	95		2 × 300 mg Clindamycin for 7 days plus(sequentially) vaginal *Lactobacillus* capsule (Gynophilus; Laboratoires Lyocentre, Aurillac Cedex, France) for 7 days. Each capsule contained at least 109 CFU of live *L. casei rhamnosus* (Lcr35), 5.59 mg lactose, and 3.41 mg magnesium stearate	2 × 300 mg Clindamycin for 7 days only		Cure rate (4 weeks)
8	Eriksson (2005) [40]	First-onset BV	127	128		Clindamycin ovules 100 mg vaginally once daily for 3 days plus (sequentially) tampons impregnated with freeze-dried *Lactobacillus gasseri*, *L. casei* var *rhamnosus* and *L. fermentum* during the following menstruation	Clindamycin ovules 100 mg vaginally once daily for 3 days plus (sequentially) placebo tampons		Cure rate (4–8 weeks)
9	Bohbot (2018) [41]	Recurrent BV	50	48		Oral metronidazole treatment 1 g/day for 7 days plus (sequentially) vaginal capsules of *L. crispatus* IP 174178 (109 CFU per gram for 14 days for two menstrual cycles	Oral metronidazole treatment 1 g/day for 7 days plus(sequentially) placebo capsules		Recurrence rate (12–16 weeks)
10	Sgibnev (2020) [42]	BV with Trichomonas Vaginalis	44	42		Metronidazole 500 mg twice a day for 7 days plus (simultaneously) one capsule of a probiotic Gynophilus® (Laboratoires Lyocentre, France) vaginally twice a day for 7 days	Metronidazole 500 mg twice a day for 7 days plus (simultaneously) one capsule of a placebo vaginally twice a day for 7 days		Cure rate(4 days)
									Cure rate (8 days)
									Cure rate (15 days)
11	Elsharkawy (2021) [43]	Recurrent BV	144	129		Clindamycin 2% vaginal cream 5 g at bedtime for 7 days plus (sequentially) continuous probiotics vaginal capsule once daily for 6 weeks	Clindamycin 2% vaginal cream 5 g at bedtime for 7 days plus (sequentially) interrupted probiotics vaginal capsule twice a week once daily for 6 weeks		Cure rate (1 month)
									Recurrence rate (1 month)
									Recurrence rate (3 months)
									Recurrence rate (6 months)
									Recurrence rate (9 months)

E = Experimental group; C1 = Control group 1; C2 = Control group 2.
